# Dental Management of a Patient with Nager Acrofacial Dysostosis

**DOI:** 10.1155/2015/984732

**Published:** 2015-10-07

**Authors:** R. Bozatlıoğlu, A. P. Münevveroğlu

**Affiliations:** Pediatric Dentistry, Faculty of Dentistry, Istanbul Medipol University, 34083 Istanbul, Turkey

## Abstract

Nager syndrome is a rare syndrome resulting from developmental abnormalities of the first and second branchial arches. Nager syndrome is rare and mostly sporadic. The main clinical features consist of craniofacial, limb, and musculoskeletal morphogenesis. These findings included malar hypoplasia, maxillomandibular hypoplasia, micrognathia, downslanting palpebral fissures, cleft palate, ear anomalies, hypoplastic thumb, short forearm, proximal radioulnar synostosis, atrial septal defect, lower limb deformities, and flat nasal bridge. The prevalence is unknown; about 100 cases of Nager syndrome have been published up to now. Patients with Nager syndrome are found worldwide among all racial and ethnic groups. Trismus and glossoptosis resulting in oropharyngeal airway narrowing cause life-threatening respiratory distress for patients with Nager syndrome. In this case report, dental rehabilitation of a 10-year-old child with Nager syndrome is presented.

## 1. Introduction

Nager syndrome is a rare syndrome resulting from developmental abnormalities of the first and second branchial arches and is mostly sporadic; however, autosomal dominant or autosomal recessive inheritance has been reported. Nager syndrome has an alteration of the 9q32 chromosome, 1q12q21 deletion. The prevalence is unknown; about 100 cases of Nager syndrome have been published up to now. Patients with Nager syndrome are found worldwide among all racial and ethnic groups [1–3].

The main clinical features consist of craniofacial, limb, and musculoskeletal morphogenesis. Oropharyngeal airway narrowing causes life-threatening respiratory distress for patients with Nager syndrome. The craniofacial abnormalities include zygomatic and maxillomandibular hypoplasia, micrognathia, downwards palpebral fissures, absence of the lower lid eyelashes, lower lid coloboma, flat nasal bridge, cleft lip and palate, short soft palate, low-set and posterior-rotated ear, ear canal atresia, lack of development of the internal and external ear, hearing problems, speech problems, and feeding problems. Respiration and feeding problems are due to the mandibular hypoplasia with micrognathia, tongue and severely restricted jaw opening. The hearing loss is due to the severity of the ear abnormalities. There may be speech problems due to the impaired hearing. The musculoskeletal abnormalities include preaxial upper-limb deformities, radial defect, radioulnar synostosis, short forearm, and absence of digits. Cardiovascular anomalies include Fallot tetralogy and/or ventricular septum defect. Nager syndrome does not affect a child's intelligence [1–4]. In these patients with restricted jaw opening, chewing is not possible, and oral hygiene is a major problem. Severe dental decay without the option of adequate treatment is very common. Owing to the hand and limb abnormalities, manipulating implements may be difficult, and self-care may not be possible [4]. In addition to enamel hypoplasia, oligodontia and dental malocclusion can be detected [1–3].

The purpose of this case report is to present oral findings and dental treatment of a patient with Nager syndrome.

## 2. Case Report

A 10-year-old boy was referred to Yeditepe University, Faculty of Dentistry, Department of Pedodontics, in Istanbul with complaint of dental caries. The patient was born to a healthy mother and healthy father at 40 weeks of gestation. The pregnancy of mother was uncomplicated. The mother's history regarding alcohol, smoking, and drug abuse was negative. Family history was not noted regarding craniofacial disorder. He has two normal sisters. A diagnosis of Nager syndrome was made around the age of 2 years when facial and limb anomalies became obvious.

**Figure 1:**
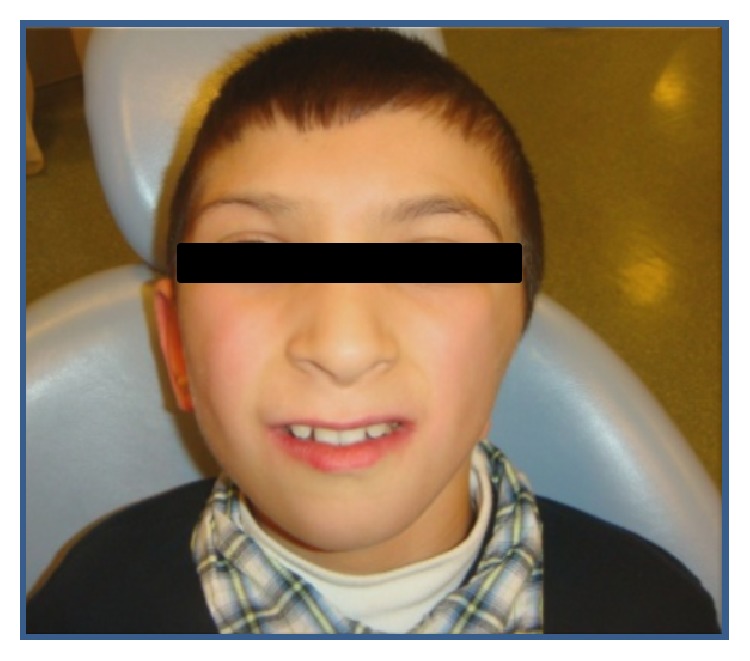
Craniofacial anomalies present included hemifacial atrophy, maxillomandibular hypoplasia, severe micrognathia, malar hypoplasia, flat nasal bridge, and left ear canal atresia.

**Figure 2:**
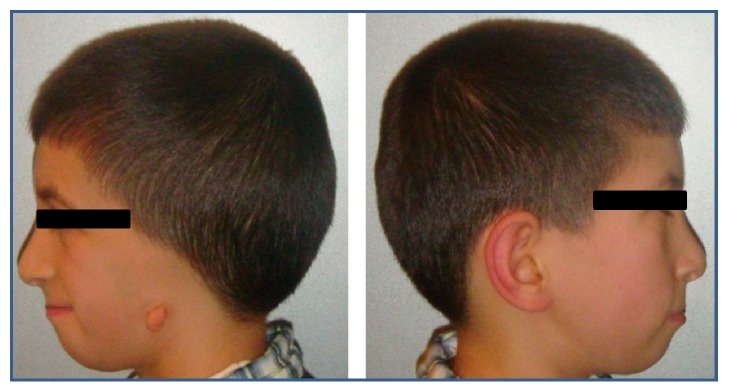
Lack of development of the internal and external ear with related hearing problems and severe mandibular hypoplasia with retrognathia.

Craniofacial anomalies present in the patient include hemifacial atrophy, maxillomandibular hypoplasia, severe micrognathia, restricted jaw opening, malar hypoplasia, flat nasal bridge, cleft palate, shortened soft palate, uvula atresia, left ear canal atresia, and lack of development of the internal and external ear (Figures 1 and 2). Other features found in the patient were respiration, feeding, and speech problems as well as short forearm and difficulty in fully extending the elbows. There was no evidence of mental retardation.

**Figure 3:**
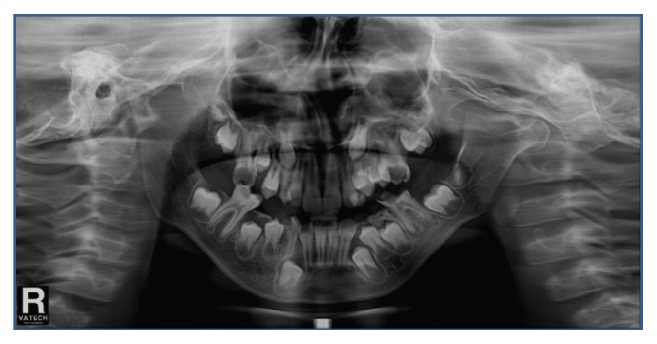
Panoramic radiograph of the patient before treatment.

**Figure 4:**
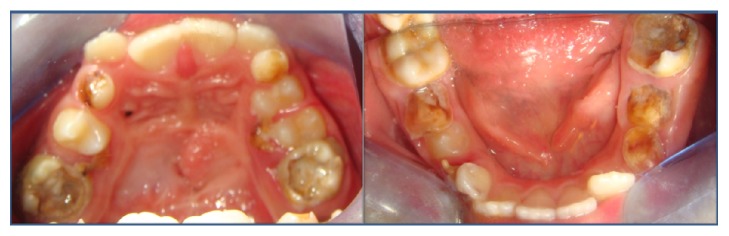
In intraoral findings, numerous carious teeth (#16, #55, #53, #26, #65, #63, #36, #75, #74, #46, #85, #84, and #83), hypomineralisation (#16, #26, #36, and #46), and cleft palate were seen.

**Figure 5:**
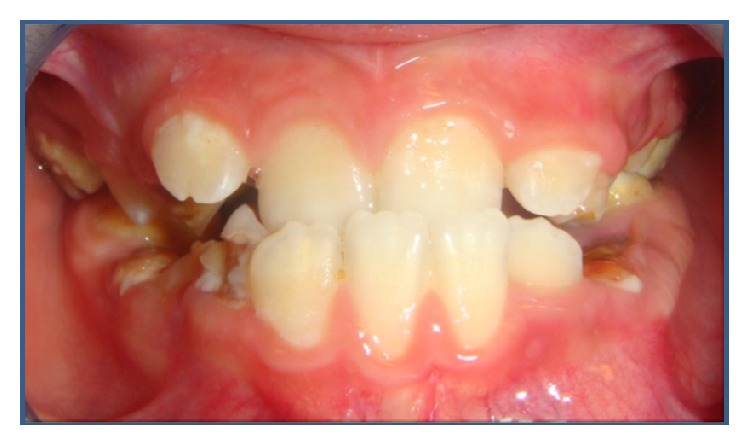
Abnormal overbite with overlap of the upper teeth.

Intraoral and radiographic examination revealed that oral hygiene is a major problem. He had numerous carious teeth (#16, #55, #53, #26, #65, #63, #36, #75, #74, #46, #85, #84, and #83) and hypomineralisation teeth #16, #26, #36, and #46, restricted mouth opening, severe maxillomandibular hypoplasia with severe micrognathia, and space deficiency in maxillary and mandibular arch (Figures 3, 4, and 5).

**Figure 6:**
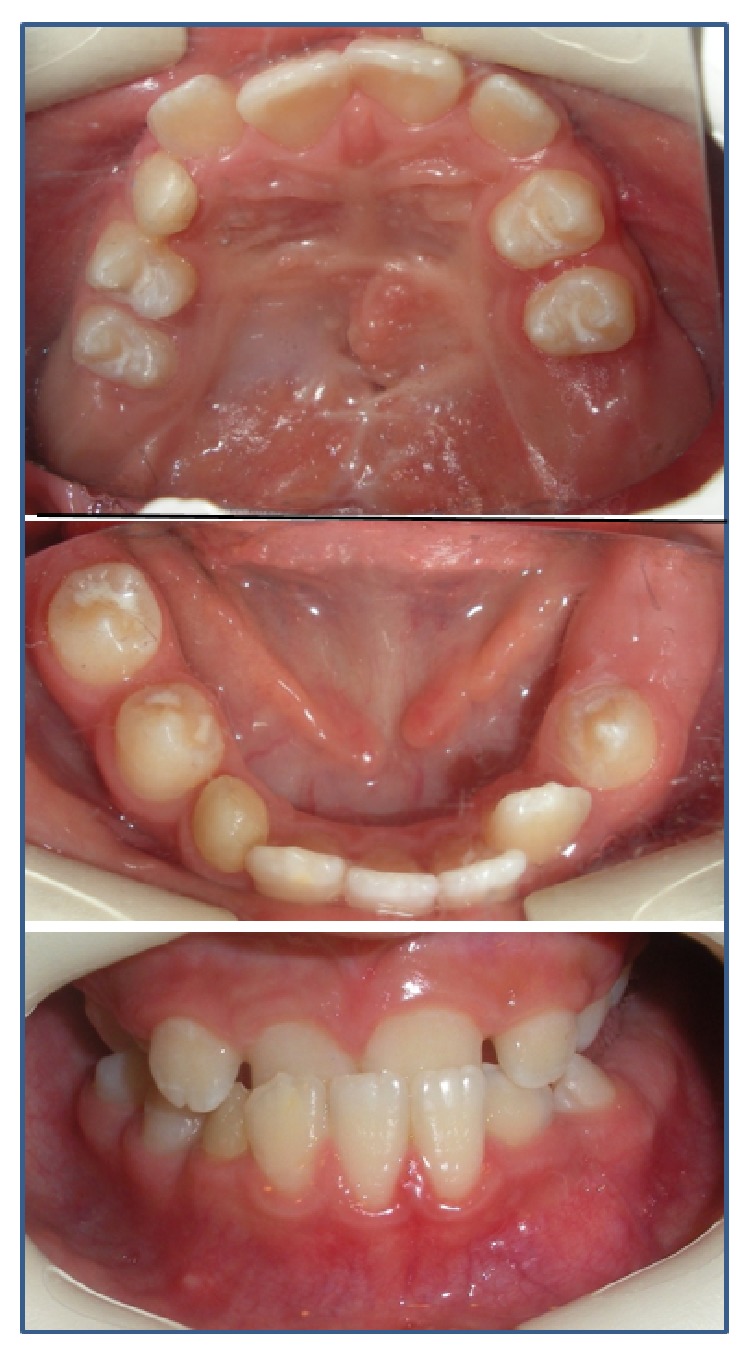
Intraoral appearance of the patient after treatment.

Pulpectomy and composite restoration was performed (#53 and #83). Teeth #16, #55, #26, #65, #63, #36, #75, #74, #46, #85, and #84 were extracted. Teeth #15, #14, #25, #24, #34, #45, and #44 were restored with fissure sealant followed by topical fluoride application (Figure 6). Oral hygiene education was given to the patient and his parents. Orthodontic consultation was held. The patient and his parents were instructed about severity of orthodontic treatment. The patient was rescheduled to visit every three months because of high caries risk.

## 3. Discussion

Most of the patients with Nager syndrome are mostly sporadic. These abnormalities can be diagnosed prenatally with the help of ultrasonography. Perinatal mortality is about 20% because of oropharyngeal airway narrowing [1, 5]. The patient was born to a healthy mother and healthy father at 40 weeks of gestation. The pregnancy of the mother was uncomplicated and she did not have any harmful habits. He has two normal sisters. In perinatal period, abnormality was diagnosed with ultrasonography but his family preferred to continue with the pregnancy. He uses nasopharyngeal airway when sleeping due to oropharyngeal airway narrowing observed at birth.

The differential diagnosis of Nager syndrome is done with Treacher-Collins, trisomy 18, Pierre-Robin, and Goldenhar syndrome. The craniofacial features of Nager syndrome resemble Treacher-Collins syndrome, including micrognathia, malar hypoplasia, downslanting palpebral fissures, and ear anomalies but mandibular hypoplasia is more severe in Treacher-Collins syndrome. Nager syndrome can be distinguished from Treacher-Collins syndrome by preaxial upper-limb deformities, such as thumb anomaly, radial defect, and radioulnar synostosis. The facial features and upper-limb anomalies are diagnostic for Nager syndrome. Trisomy 18 is usually associated with clinodactyly, not with absence of digits [1, 6]. In this case report, the patient had exactly similar features with Nager syndrome.

Mandibular malformations, missing joint structures, restriction in jaw movements, and limb abnormalities cause oral hygiene deficiency. In this case report, the patient had limited mouth opening and multiple carious teeth. Restricted mouth opening also made dental treatment extremely difficult and requires treatment under general anaesthetic. Treatment under general anaesthesia can be challenging in Nager syndrome because of oropharyngeal airway narrowing. Severe airway obstruction is derived from mandibular hypoplasia with micrognathia, retroplaced tongue, and strabismus. Moreover, hearing and speech difficulty lead to communication problems with patients [7]. The patient has mandibular hypoplasia and severe micrognathia. He had three general anesthesia attempts for treatment of oropharyngeal airway narrowing but one of the three was achieved by surgical and anaesthetic team. Moreover he has hearing and speech problems but intelligence is of normal level. In this case report, dental treatments of the patient were performed under local anesthesia.

## 4. Conclusion

Nager syndrome is a rare disorder which inhibits treatment under general anaesthesia because of oropharyngeal airway narrowing. It is important that these patients receive comprehensive prevention and regular dental review in an effort to improve their oral health and therefore avoid, when possible, the need for dental treatment under general anaesthesia. Patients with Nager syndrome should be referred to pedodontics for oral hygiene instruction, dietary advice, and fluoride prevention.
